# 

**Published:** 1916-05-06

**Authors:** 


### CONTENTS.

| | PAGE |
|---|---|
| **Some Messages of the War** ... ... ... ... | 115 |
| **The Tragic Tale of Wittenberg** ... ... ... ... | 116 |
| Shall there be Medical Reprisals? | |
| **Hospital and Institutional News** ... ... ... | 117 |
| Decorations for Consultants with the Army—The Order of St. Michael and St. George—"And some there be which have no Memorial"—Shakespeare on Medicine—The R.A.M.C. and Civilian Sick—A Scottish Hero—A Hospital Project to be Defeated—Old Hospital Life in Norwich—A Hospital without a Treasurer—Disappointed Bognor—A Clever Escape from an Asylum—The Boy and His Future—Death under an Anæsthetic at the Anti-Vivisection Hospital—An Appreciated Return to Duty—Mr. F. H. Bentham's Review of Poor-Law Progress—Butter *v.* Margarine—This Week's Drug Market. | |
| **Sidelights on Hospital History** ... ... ... | 122 |
| The Norfolk and Norwich Hospital, 1771-1847. | |
| **Insanity and Temperament** ... ... ... | 123 |
| I.—Mediæval *v.* Modern Ideas in Medicine. | |
| **The State Endowment of Motherhood** ... | 124 |
| **The Institutional Treatment of Consumption** | 125 |
| I.—The Sorting of the Patients. | |
| **How to Run a Laundry** ... ... ... ... | 126 |
| II.—The Week's Work. | |
| **A Thames-side Military Hospital** ... ... | 127 |
| St. Thomas's and the War. | |
| **Parliament and the Profession** ... ... ... | 128 |
| **Edward Livingston Trudeau, M.D., and R. L. Stevenson** ... ... ... ... ... ... ... | 129 |
| Fellow-sufferers from Tuberculosis. | |
| **The Institutional Library** ... ... ... ... | 130 |
| How to Live Long—Health for Young and Old—Modern Medicine and Some Modern Remedies—Nervous Asthma: Its Pathology and Treatment—School Children: Their Care and Nursing—The Eyes of Our Children—Cerebro-Spinal Fever—Cleft-Palate and Hare-Lip—The Care of the Body. | |
| **The Matrons' and Sisters' Department** ... | 133 |
| Nursing in the United States. | |
| State Registration in America. | |
| Round the Hospitals. | |
| Hospital Children's Garments—Training at Westminster—War Influences at Salisbury—Imperial Nurses' Club. | |
| **Hospital Architecture and Construction** ... | 135 |
| Toronto General Hospital: Accommodation 700 Patients. | |
| **The Editor's Letter-Box** ... ... ... ... ... | 138 |
| St. George's Hospital. | |
| **Personalia**... ... ... ... ... ... ... | 138 |
| **Matrons' and Sisters' Appointments** ... ... | 138 |
| **Hospital Results in 1915** ... ... ... ... | xii |
| More Early Reports of the Voluntary Hospitals. | |
| **Passing Events and Topics** ... ... ... ... ... | xii |
| **The Institutional Worker** ... ... (Suppl.) | 1 |
| Poor-Law Infirmary to Military Hospital. | |

